# Oral/enteral fluid resuscitation in the initial management of major burns: A systematic review and meta-analysis of human and animal studies

**DOI:** 10.1016/j.burnso.2024.100364

**Published:** 2024-11

**Authors:** Kai Hsun Hsiao, Joseph Kalanzi, Stuart B. Watson, Srinivas Murthy, Ani Movsisyan, Kavita Kothari, Flavio Salio, Pryanka Relan

**Affiliations:** a Emergency Medical Teams, Country Readiness Strengthening Department, World Health Organization, Geneva, Switzerland; b Department of Anaesthesia, Critical Care and Emergency Medicine, College of Health Sciences, Makerere University, Kampala, Uganda; c Canniesburn Plastic Surgery and Burns Unit, Glasgow Royal Infirmary, Glasgow, United Kingdom; d Faculty of Medicine, University of British Columbia, Vancouver, Canada; e Consultant to the Methods and Standards Team, World Health Organization, Geneva, Switzerland; f Consultant to Library and Digital Information Networks, World Health Organization, Geneva, Switzerland

**Keywords:** Burns, Fluid therapy, Intravenous fluid, Mass casualty incidents, Oral fluid, Oral rehydration solution

## Abstract

**Background:**

Timely and safe intravenous (IV) fluid resuscitation for major burns may be difficult or impossible during mass casualty burn incidents. Oral/enteral fluid resuscitation may be an alternative.

**Objectives:**

To synthesize and assess certainty of evidence on oral/enteral fluid resuscitation as compared to IV or no fluid resuscitation for major burns.

**Methods:**

PubMed, EMBASE, CINAHL, and Cochrane Library were searched on 8 September 2023. Primary quantitative studies meeting criteria as assessed by two reviewers were included. Meta-analyses for outcome effects of oral/enteral versus IV and of oral/enteral versus no fluid resuscitation were conducted. Evidence certainty was assessed using GRADE.

**Results:**

Seven human and eight animal studies were included. Three human RCTs totalling 100 participants contributed to estimates. Compared to IV fluid resuscitation, oral/enteral fluid resuscitation is associated with a statistically insignificant increased risk of mortality (OR 1.33, 95% CI 0.33–5.36) but the evidence is very uncertain, and no difference in urine output (SMD −0.17, 95% CI −0.65–0.31) with moderate certainty of evidence. Eight controlled animal studies totalling 212 participants contributed to estimates. From these animal studies, enteral fluid resuscitation may increase mortality (OR 36.00, 95% CI 2.72–476.28), worsen creatinine levels (MD 22 mmol/L, 95% CI 15.8–28.2), and increase urine output (MD 1 ml/kg/h, 95% CI 0.55–1.45) compared to IV, but all with very low certainty of evidence. Again, from animal studies, all the evidence is very uncertain, but compared to no fluid resuscitation, enteral resuscitation is associated with a statistically insignificant reduction in mortality (OR 0.29, 95% CI 0.08–1.09), improved creatinine levels (SMD −3.48, 95% CI −4.69 to −2.28), and increased urine output (MD 0.55 ml/kg/h, 95% CI 0.38–0.72).

**Conclusions:**

Current evidence comparing oral/enteral and IV fluid resuscitation for major burns is limited and uncertain. However, where IV fluid resuscitation is unavailable or delayed, oral fluid resuscitation could be considered.

## Highlights

• Effect of oral/enteral versus IV fluid resuscitation for major burns is still uncertain.
• Oral fluid resuscitation may be considered in mass casualty burns if IV unavailable.
• Research on optimal fluid and efficacy limits for oral fluid resuscitation is needed.

## 1 Introduction

Timely and adequate fluid resuscitation is fundamental to the initial management of patients with major burns. The intravenous (IV) route for fluid resuscitation is the established standard in modern burns care due to its directness and titratability for expanding circulating volume [1]. However, there are scenarios, such as burn mass casualty incidents (MCI), where the safe administration and monitoring of IV fluid resuscitation for burns may be unachievable or significantly delayed due to patient numbers overwhelming available skilled staff, supplies (such as IV cannulas and fluid bags) and/or logistic capacities [2], [3]. In such scenarios, it may be necessary to consider alternative routes of administration for initial fluid resuscitation.

Oral and other enteral routes, such as gastric or rectal infusions, for fluid resuscitation in burns have been considered as early as the mid-20th century, primarily driven by concerns for mass casualties from nuclear warfare at the time. The 1950 Surgery Study Section of the National Institutes of Health recommended “that the use of oral saline solution be adopted as standard procedure in the treatment of shock due to burns and other serious injuries in the event of large-scale civilian catastrophe”, although acknowledging that further research is required [4]. Contemporary recommendations for oral fluid resuscitation as a contingent option to IV fluids remain in various guidelines for burns care in disaster, austere, low-resource and combat casualty prolonged field care settings [3], [5], [6], [7], [8]. This includes a recommendation for burns care in MCIs published in 2020, which advocated: “in the event of an MCI, oral fluid should be encouraged (as appropriate) on-scene. Burn injured patients should not routinely receive IV burn resuscitation fluid at the scene” [9].

Studies, predominantly in animal models but also human clinical studies, have indicated feasibility of oral/enteral fluid resuscitation for burns in terms of, for example, intestinal absorption, hemodynamics, reduced kidney injury, maintained urine output and clinical implementation [10], [11], [12], [13]. None have been sufficient for definitive guidance for clinical practice, and substantial uncertainties remain around aspects such as efficacy limits, applicability to specific patient populations, effects of altered gastrointestinal function in burn shock, vomiting, and aspiration risk [10], [11], [12], [13]. Two previous non-systematic reviews by *Cancio et al.* in 2006 and by *Kramer et al.* in 2010 concluded that although more research is required, the available studies suggested that the enteral route for fluid resuscitation can be feasible and effective, leading to their recommendation of considering oral resuscitation with salt-containing fluids for conscious patients that have no apparent gastrointestinal injuries if IV fluid resuscitation were unavailable or delayed [1], [2]. However, there have been no systematic reviews of the available evidence to date.

The purpose of this review was to synthesize and assess the certainty of available evidence on oral/enteral fluid resuscitation for major burns. This was part of a process to inform a current World Health Organization (WHO) Guideline Development Group (GDG) in their development and update of guidelines for burns care in MCIs. An earlier systematic review by the authors had looked at oral/enteral fluid resuscitation in the specific setting of MCIs, and yielded no studies (unpublished, PROSPERO study protocol registration #CRD42023430738). This current review broadened the approach and sought the available evidence from any human clinical setting and from animal studies. The structured review question was:

**P** For patients with major burns (≥15% total body surface area (TBSA) for adults and ≥10% TBSA for children),

**I** does oral/enteral fluid resuscitation for initial management (in the first 24–72 h post-burn injury)

**C** compared to (i) IV fluid resuscitation, or to (ii) no fluid resuscitation for initial management

**O** affect outcomes of mortality, adequate urine output, acute kidney injury (AKI), complications of fluid over-resuscitation, or aspiration events?

## 2 Materials and methods

A study protocol was developed and registered on PROSPERO (registration #CRD42023462845). The review conforms to the Preferred Reporting Items for Systematic Reviews and Meta-Analyses (PRISMA) guidelines [14].

### 2.1 Review question formulation and definitions

The review topic was initially identified by the GDG. Subsequently, the review question was formulated by the authors, and affirmed by the GDG.

The population of interest were patients with burn injury of a severity that would indicate for fluid resuscitation (i.e. major burn). The lower-end thresholds that typically define this are TBSA ≥15% in adults and ≥10% in children, as specified in some guidelines [6], [15], [16]. This lower-end definition was adopted for this review to ensure broader capture of relevant studies.

The focus of this review was on initial fluid resuscitation. This was objectively defined as the fluid therapy commenced in the first 24–72 h post-burn injury. This corresponds to the acute phase of burn injury characterized by burn shock (with peak at 12–24 h), representing the critical period for fluid resuscitation [17]. For the purposes of this review, oral/enteral fluid resuscitation primarily referred to fluids administered by mouth, but also included gastric/jejunal tube infusion; enteral fluid resuscitation via the rectal route (proctoclysis) was excluded as this was not an intended intervention of interest.

The outcomes of interest were selected based on prioritization by the GDG as per WHO protocol [18]. These had included pulmonary edema and abdominal compartment syndrome as separate outcomes, but were subsequently grouped together under complications of fluid over-resuscitation.

### 2.2 Search strategy

The search strategy was developed and conducted by a health information specialist (KK) on the following databases: PubMed, EMBASE, CINAHL, and Cochrane Library. Search strings were adjusted as appropriate for each database and included the following search terms and appropriate synonyms: (burn OR thermal injury) AND (fluid management OR fluid resuscitation OR fluid therapy) AND (oral OR enteral) and NOT (enteral-nutrition OR feeding). No filters or limits were applied. The search was conducted on 8 September 2023. An example search strategy is provided in Appendix.

Additionally, a Google search using the primary search terms “burn”, “oral” and “fluid resuscitation” as well as reference search of identified studies, reviews and relevant published guidelines were conducted.

Search results were exported into the Covidence systematic review program (Veritas Health Innovation Ltd., Melbourne, Australia) where records were deduplicated.

### 2.3 Eligibility criteria and study selection

The pre-defined study inclusion criteria were:

1. Primary, empirical studies with quantitative data, including case reports and case series; both human and animal studies were eligible
2. Study population were those with major burn injuries (i.e. ≥15% TBSA in adults or ≥10% TBSA in children, and ≥15% TBSA for animal studies)
3. Study reported on the use of oral/enteral fluid resuscitation in the initial management (defined as management in the first 24–72 h post-burn injury)
4. Study included outcomes in terms of mortality, urine output, AKI, complications of fluid over-resuscitation (such as pulmonary edema and abdominal compartment syndrome), or aspiration events
5. Written in English

The exclusion criteria were:

1. Editorials, commentaries, study protocols and modelling studies
2. Studies that did not report on the use of oral/enteral fluid resuscitation in the initial management of major burns
3. Studies that reported on enteral resuscitation via the rectal route only
4. Studies that did not include data on any of the outcomes of interest

Studies that did not have a comparator group, that is, single-armed intervention studies of oral/enteral fluid resuscitation that otherwise fulfilled inclusion criteria were not excluded from the review. However, these would not be amenable to nor included in the main syntheses and meta-analyses of intervention effect.

Following removal of duplicates, title and abstract screening and subsequent full-text review were independently conducted by two reviewers (KH and JK) according the above criteria to identify eligible studies. In the event of disagreement, a third reviewer (PR) was available to adjudicate.

### 2.4 Data extraction

Data was extracted on Covidence using a pre-specified standardized form. For each study, data was extracted on: Study characteristics (author, year, title, study type/design, setting and country); study population (inclusion/exclusion criteria, sample size, age, type(s) of burn injury, severity of burn, sub-groups within study); intervention characteristics (timing, formulation, administration, rate, duration, total volume and adjuncts for oral fluid); outcomes (mortality rate, urine output, occurrence of AKI, complications of fluid over-resuscitation and aspiration events); and any reported constraints or challenges for oral fluid resuscitation. Data was independently extracted by two reviewers (KH and JK) and final consensus achieved through discussion. In the event of disagreement, a third reviewer (PR) was available to adjudicate.

### 2.5 Risk of bias assessment

Risk of bias assessments were independently conducted by two reviewers (KH and JK) using the revised Cochrane Risk-of-Bias tool for randomized trials (RoB 2) [19] or the JBI Critical Appraisal Checklist for Cohort Studies [20], depending on study design. In the event of disagreement, a third reviewer (PR) was available to adjudicate. Risk of bias figures were generated using the Risk of Bias Visualization (robvis) tool (McGuinness, LA, et al., Bristol, UK) [21].

### 2.6 Data synthesis and statistical analysis

Results from human and animal studies were synthesized separately. Separate analyses were conducted for each of the two review question comparisons: oral/enteral versus IV fluids, and oral/enteral versus no fluids. Intervention effect estimates were summarized using odds ratios (with corresponding 95% confidence intervals (CI)) for dichotomous outcomes (mortality, AKI, complications of fluid over-resuscitation and aspiration events), and mean differences (with corresponding 95% CI) for continuous outcomes (urine output and serum creatinine levels). For the animal studies, the effects on renal function as measured by serum creatinine levels were analysed instead of occurrence of AKI as this was the only way in which data related to this outcome was presented. Pooled effect estimates were calculated using a random-effects model with the Mantel–Haenszel method for dichotomous outcomes and the DerSimonian and Laird method for continuous outcomes. Significance was set with p-value <0.05 and 95% CI. Substantial heterogeneity was considered for *I^2^* values ≥50%. Meta-analysis and forest plot generation were conducted using Cochrane Review Manager (RevMan) Web version 7.4.0 (Cochrane Collaboration, London, UK).

Sub-group analyses for children (age <18 years old), by burn type, by burn severity (TBSA <40% versus TBSA ≥40%) and for those with inhalational injuries had been included in the study protocol. These sub-groups were identified by the GDG as clinically important. However, due to a paucity of studies, small number of participants, and lack of stratification by some of these characteristics in reported data, no sub-group analyses were performed.

### 2.7 Certainty of evidence assessment

Certainty of evidence was assessed using the GRADE approach [22]. Two reviewers (KH and JK) independently rated the risk of bias, inconsistency, indirectness, publication bias and imprecision for each comparison and outcome that had effect estimates. In the event of disagreement, a third reviewer (PR) was available to adjudicate. The overall certainty of evidence for each outcome and the summary of findings tables were generated using the GRADEpro Guideline Development Tool program (McMaster University and Evidence Prime Inc., Hamilton, ON, Canada).

## 3 Results

**Fig. 1:**
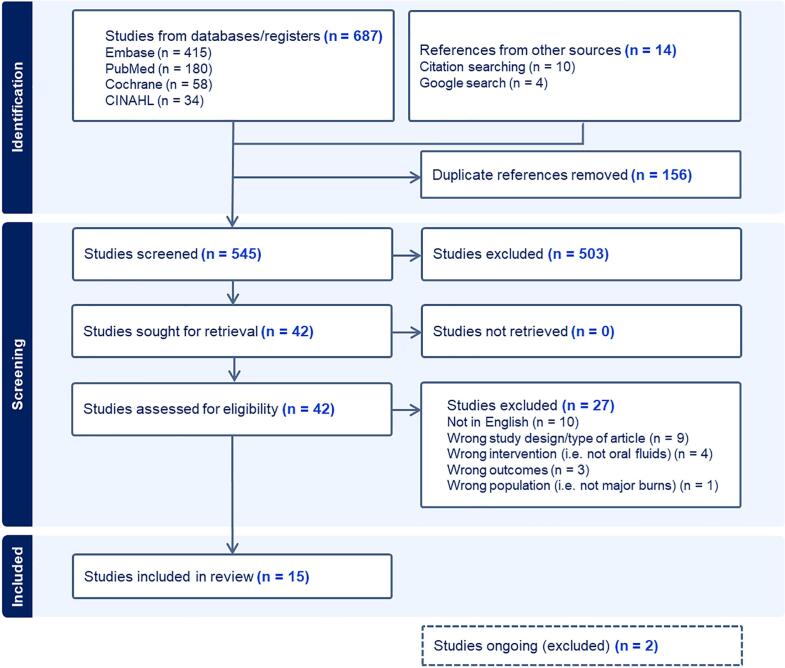
PRISMA flow diagram for study selection.

The search strategy yielded 701 records. Following deduplication, screening, and full-text review, 15 studies were included in this review. Fig. 1 shows the PRISMA flow diagram for study selection, including exclusion reasons. Of the 15 included studies, seven were human studies (Table 1) [12], [13], [23], [24], [25], [26], [27] and eight were animal studies (Table 2) [10], [11], [28], [29], [30], [31], [32], [33].

**Table 1:** Included human studies, n = 7.

Author, year, country		Study population							
	Study design	N	Age (range)	%TBSA (range)	Exclusion criteria	Intervention	Comparison	Reported outcome(s) relevant to review	Key findings
**Mehta,** 2021, Nepal	RCT	30	Median: 49 yrs IQR: 30.5–59.25 yrs	20–40%	(1) Electrical burns, chemical burns, inhalation injury (2) Patients in overt shock (3) Pregnant, psychiatrically unstable (4) Patients with oropharyngeal defects and/or previously known diagnoses leading to high risk of aspiration, and/or precluding safe nasal-enteric access (5) Patients with history of chronic nausea and/or vomiting, including those with a diagnosis of gastroparesis due to diabetes (6) Patients with a Baux score of over 100 (age + TBSA), patients with declared palliative intent on admission, and patients for whom clinicians have high level of concern based on clinical judgement	ORS (n = 15) (administered by mouth or NG; supplemental IV fluid if needed as per protocol)	IV Ringer’s Lactate (n = 15) (given according to “standard of care”)	• Mortality [in-hospital] • Urine output [hourly average for 24hrs] • AKI [signs of AKI in 72hrs]	Pilot feasibility study. 9/15 (60%) of oral fluid group crossed over to IV fluid resuscitation due to GI intolerances such as nausea or vomiting.
**Moghazy,** 2016, Egypt	RCT	30	15–55 yrs	15–55%	(1) Patients with co-morbidities (diabetes mellitus, hypertension, renal failure, ischemic heart disease & chronic liver disease) (2) Inhalation injury (3) Associated injuries (multiple trauma, internal hemorrhage etc.) (4) Pregnancy and lactation	WHO ORS (Rehydran-n) + 5 g salt tablet hourly (n = 10) (administered via NG; 150–200 ml/kg over first 24hrs; allowed to drink freely with any additional fluids)	IV Ringer’s Lactate (n = 20) (4 ml/kg/%TBSA over first 24hrs)	• Urine output [hourly average in ml/kg/hr for 72hrs]	No significant difference in assessed outcomes, including urine output, between study (oral) and control (IV) groups.
Milner, 2011, United States	Descriptive cohort	3	31–48 yrs	20–40%	None specified	IV Ringer’s lactate + oral Ceralyte (n = 3)	−-	• Urine output [adequacy]	Oral fluid was able to replace 57.4–77.5% of calculated IV fluid requirements.
**El-Sonbaty,** 1991, Egypt	RCT	40	4 months − 12 yrs	10–20%	None specified	WHO ORS (Rehydran-n) (n = 20) (4 ml/kg/%TBSA over first 24 hrs)	IV solution not specified (n = 20) (given according to Parkland formula)	• Mortality [in-hospital] • Urine output [mean ml/hr for 24hrs]	Encouraging results from use of oral resuscitation of moderately burned children. Hyponatremia occurred in both groups.
Monafo, 1970, United States	Descriptive cohort	10	2–70 yrs	22–95%	None specified	IV+oral hypertonic lactated saline (n = 10)	−-	• Mortality [at 24hrs, total] • Urine output [total ml/24hrs for 48hrs] • AKI • Over-resuscitation [signs of excess pulmonary fluid]	Hypertonic lactated saline solution proved simple, safe, and efficacious for resuscitation of the severely burned patient. In all patients, urine volumes were adequate. Signs of excess pulmonary fluid were not observed.
Sorensen, 1968, Denmark	Descriptive (serial) cohorts	69	Not reported	Not reported	(1) Transferred patients already commenced on serum therapy or other therapy (2) Patients for which any chance of survival was out of the question and so no treatment was given	Oral fluid + salt tablet +/- IV saline (n = 26) (drank ad libitum; any type of fluid e.g. tea, beer, milk, water etc.)	IV dextran + oral fluids (n = 43) (120 ml/%TBSA with half in first 8hrs, quarter in next 16hrs and rest in next 24hrs)	• Mortality [at 48hrs, total] • Urine output [mean ml/hr for first and second 24hrs] • AKI [anuria]	80% of patients with burns not exceeding 45% were able to drink so liberally that this was their only anti-shock therapy.
Markley, 1956, Peru	RCT	193	2 months − 68 yrs	≥10%	(1) Burns involving less than 10% of body surface area (2) Pulmonary burns (3) Age over 68 years (4) Burns not treated within eight hours (5) Cases in which death occurred within two hours after treatment had begun	Oral and/or IV saline (n = 100) (given via NG for infants < 2 yrs)	IV colloid (plasma, blood, or povidone) + oral sucrose in water (n = 93) (given according to Evans formula)	• Mortality [at 48hrs, total] • Urine output • Over-resuscitation [generalized or pulmonary edema]	No significant difference was found between the two groups (saline versus colloid) as to 48-hr or ultimate total mortality.

**Table 2:** Included animal studies, n = 8.

Author, year, country		Study Population						
	Study design	N	Species	%TBSA	Intervention(s)	Comparison(s)	Reported outcome(s) relevant to review	Key findings
Burmeister, 2018, United States	Controlled study	24	Pig/swine	40%	Enteral ORS WHO formula (15 ml/kg/d) (n = 6) Enteral ORS WHO formula (70 ml/kg/d) (n = 6)	IV Ringer’s lactate (15 ml/kg/d) + enteral ORS (n = 6) IV Ringer’s lactate (Modified Brooke formula) + enteral ORS (n = 6)	• Urine output [mean ml/kg/hr for 48hrs]	Enteral resuscitation after burn injury provided therapeutic benefit. Incorporating enteral fluids may reduce IV fluid volumes.
**Gomez,** 2018, United States	Controlled study	18	Pig/swine	40%	Enteral ORS WHO formula (70 ml/kg/d) (n = 6) Ad libitum water (n = 6)	No fluid (n = 6)	• Mortality [at 48hrs] • Urine output [mean ml/kg and ml/kg/hr for 48hrs] • AKI [mean serum Creatinine at 48hrs]	Enteral resuscitation with ORS rescued kidney function following burn injury, and increased urine output compared to water and no water.
**Liu,** 2018, China	Controlled study	80	Rat	50%	Enteral ORS WHO III formula (n = 20) Enteral ORS with pyruvate (n = 20) (volume/rate in accordance with Parkland formula)	No fluid (n = 20) Sham scald group (n = 20)	• Mortality [at 24hrs]	Survival was markedly improved in both enteral ORS groups; ORS with pyruvate conferred higher survival at 24 h.
**Gomez,** 2017, United States	Controlled study	25	Pig/swine	40%	Enteral ORS (15 ml/kg/d) (n = 6) Enteral ORS (70 ml/kg/d) (n = 6) Ad libitum water (n = 6)	No fluid (n = 7)	• AKI [serum Creatinine elevation]	Water restriction exacerbated burn-induced acute kidney injury. ORS and ad libitum water were sufficient to alleviate acute kidney injury.
**Liu,** 2016, China	Controlled study	40	Dog	50%	Enteral ORS, citrate-enriched (n = 10) Enteral ORS, pyruvate-enriched (n = 10) (volume/rate in accordance with Parkland formula)	IV Ringer’s lactate (Parkland formula) (n = 10) No fluid (n = 10)	• Mortality [at 24hrs, 48hrs] • Urine output [mean ml/24hrs for 48hrs] • AKI [mean serum Creatinine at 24hrs, 48hrs]	Survival at 48 hrs was highest for IV group; Enteral ORS groups had higher survival compared to no fluid. Pyruvate-ORS was superior to citrate-ORS in improving hemodynamic parameters, organ function and survival.
**Hu,** 2012, China	Controlled study	18	Dog	35%	Enteral hypertonic solution (n = 6) Enteral hypertonic solution plus mosapride (n = 6)	IV 0.9% NaCl + 5% glucose (Parkland formula) (n = 6)	• Mortality [at 6hrs] • Urine output	Enteral hypertonic electrolyte-glucose solution with addition of mosapride accelerated gastric emptying and achieved outcomes similar to IV fluids.
**Hu,** 2011, China	Controlled study	20	Dog	35%	Enteral glucose saline solution (n = 8) Enteral glucose saline solution with carbachol (n = 8) (volume/rate in accordance with Parkland formula)	No fluid (n = 10)	• Mortality [at 24hrs, 5 days]	Carbochol elevated rate of gastric emptying and intestinal water absorption. 5-day mortality was zero for carbachol group compared to other enteral and no fluid groups.
**Michell,** 2006, United States	Controlled study	11	Pig/swine	40%	Enteral ORS WHO formula (20 ml/kg/hr) (n = 5)	IV Ringer's lactate (Parkland formula) (n = 6)	• Mortality [at 4.5hrs] • Urine output [mean ml/kg/hr for 4.5hrs]	Intestinal absorption rates after burn injury were sufficient to resuscitate a 40% TBSA burn. Enteral and IV resuscitation showed equivalent effects.

## 4 Results from human studies

### 4.1 Study characteristics

Of the seven human studies, four [12], [13], [23], [24] were conducted within the last 35 years while the remaining three [25], [26], [27] were from 1970 or earlier. Two studies were conducted in the United States, two in Egypt, and one each in Denmark, Nepal and Peru. All studies were hospital-based, including five [12], [13], [23], [25], [26] where a burns unit setting was specified. Age of study participants ranged from 2 months to 70 years old, with one study [12] including only children. All burn injuries were thermal. Burn severity ranged from 10 to 95% TBSA, encompassing children and adults, with three studies [12], [23], [24] limited to moderate-severity burns ≤40% TBSA.

Oral rehydration solution (ORS) was the most commonly used fluid for oral/enteral resuscitation, and was the oral fluid used in all studies conducted within the last 35 years [12], [13], [23], [24]. Other studies used oral saline or any fluid, such as milk or water. Supplementation of oral fluids with salt tablets was used in two studies [13], [26]. IV fluid resuscitation was with Ringer’s lactate in three studies [13], [23], [24], with remaining studies using other types of crystalloid solutions and colloids, such as dextran, plasma and blood.

In terms of study design, two of the included studies were single-armed, descriptive cohort studies where combined oral and IV routes were used to demonstrate feasibility of oral fluid in supplementing IV fluids [24], [25]. Additionally, the study by *Sorensen* [26] was also considered a descriptive cohort even though it included two groups: these were two series from different time periods with patients from January 1963 to May 1964 treated with oral fluid and salt tablets with or without IV saline, and patients from May 1964 to May 1966 treated with IV dextran with oral fluids. This study was not amendable to clear comparison of oral versus IV fluids. Four of the included studies were randomized controlled trials (RCTs) [12], [13], [23], [27]. However, the RCT by *Markley et al.* [27] compared those treated with saline solutions, mainly by mouth, to those treated with IV colloid, supplemented by oral sucrose in water; there was no data stratification to enable comparison of those resuscitated exclusively via the oral/enteral versus IV routes. Due to the inability to make clear comparisons between oral/enteral and IV fluid resuscitation in the three descriptive cohort studies [24], [25], [26] and the RCT by *Markley et al*. [27], these studies were not included in the analyses of effect estimates nor GRADE assessment.

Ultimately, only three RCTs [12], [13], [23] distinctly compared oral/enteral versus IV fluid resuscitation. These three studies totalled 100 participants with age range from 4 months to 59 years old, and burn severities from 10 to 55% TBSA. Exclusion criteria were specified in two studies [13], [23], which excluded those with inhalational injuries, pregnancy, comorbidities, high aspiration risk, associated injuries such as multiple trauma, or overt shock. ORS was used for oral/enteral fluid resuscitation and Ringer’s lactate (where specified) for IV fluid resuscitation.

### 4.2 Risk of bias within studies

The assessed risk of bias within studies for RCTs and descriptive cohort studies are presented in Fig. 2A and Fig. 2B, respectively.

**Fig. 2A:**
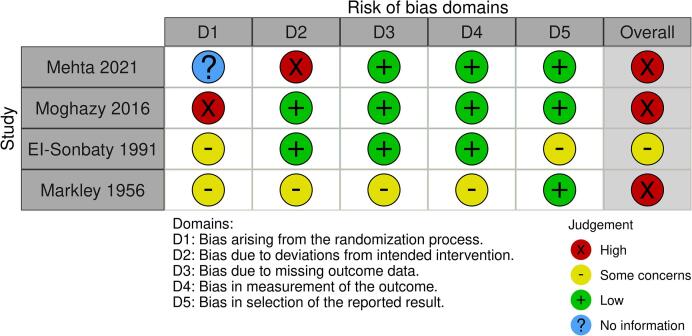
Assessed risk of bias within RCTs (human studies) using RoB 2 tool.

**Fig. 2B:**
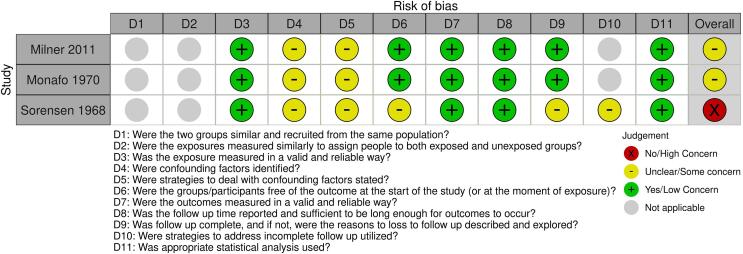
Assessed risk of bias within cohort studies (human studies) using JBI Appraisal Checklist.

Of the three RCTs that compared oral/enteral versus IV fluids, two were assessed to have high overall risk of bias: For *Mehta et al.* [23]*,* this was primarily because of the high proportion (60%, 9/15) of the oral/enteral fluid group crossing over to IV fluid resuscitation due to gastrointestinal intolerances, thus deviating from intended interventions. For *Moghazy et al.* [13], high concern was due to the randomization process with a high rate of patients refusing allocation to the oral/enteral fluid group, resulting in unequal group sizes. The RCT by *El-Sonbaty et al*. [12] was assessed as having some concerns due to use of an alternating and non-concealed allocation process.

### 4.3 Effects of oral/enteral versus IV fluid resuscitation

Mortality: Two studies [12], [23] contributed to the meta-analysis of effect on mortality with a total of 70 participants (Fig. 3A). The pooled effect estimate found a slight increased risk of in-hospital mortality with oral/enteral fluid resuscitation compared with IV (OR 1.33, 95% CI 0.33–5.36, p=0.69) but this was not statistically significant and the wide 95% CI included no effect.

**Fig. 3A:**
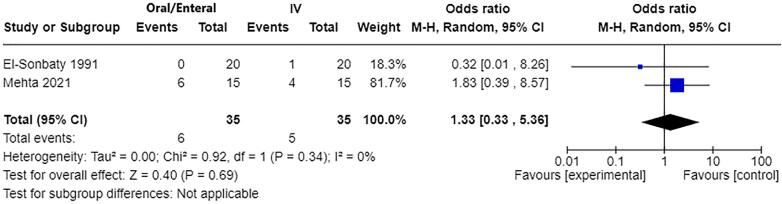
Forest plot of oral/enteral versus IV fluids (human studies) for outcome: Mortality (in-hospital).

AKI: Only one study [23] contained comparative data on AKI occurrence. However, comparative effect size was not able to be estimated due to zero events of new AKI during first 72 h post-burn injury in the IV (comparison) group (0/13, 0%) compared to one event (1/11, 9%) in the oral/enteral (intervention) group.

Urine output: Two studies [12], [13] contributed to the meta-analysis of effect on urine output with a total of 70 participants (Fig. 3B). There was little to no difference in mean hourly urine output over the first 24 h with oral/enteral fluid resuscitation compared to IV (mean standard difference −0.17, 95% CI −0.65–0.31, p=0.50).

**Fig. 3B:**
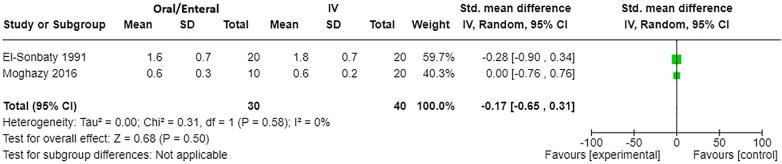
Forest plot of oral/enteral versus IV fluids (human studies) for outcome: Urine output (mean hourly urine output during first 24 h).

Complications of fluid over-resuscitation: No data to estimate effect.

Aspiration events: No data to estimate effect.

### 4.4 Effects of oral/enteral versus no fluid resuscitation

There were no human studies comparing oral/enteral versus no fluid resuscitation.

### 4.5 Certainty of evidence

Using the GRADE approach, the certainty of the above-estimated effects of oral/enteral fluid resuscitation on the outcomes of mortality and AKI was rated as very low, i.e. very uncertain. Downgrading was due to: (a) serious risk of bias due to the high overall risk of bias found in the predominating study; (b) serious indirectness as the evidence derived from these clinical trial settings of individual care in burns units may not directly apply to burn MCI settings where there will likely be less clinical attention per patient, greater delays to presentation and interventions, and more complex case-mix of patients with other associated trauma, inhalational injuries, and co-morbidities; and (c) serious imprecision with few events and a wide confidence interval. There was moderate certainty on the little to no difference in urine output with oral/enteral fluid resuscitation, with certainty of evidence downgraded for indirectness. A summary of findings with plain language descriptions of effects and certainty of evidence is presented in Table 3.

**Table 3:** Summary of Findings table for oral/enteral compared to IV fluid resuscitation for initial management of major burns (human studies).

Outcomes	Anticipated absolute effects* (95% CI)		Relative effect (95% CI)	№ of participants (studies)	Certainty of the evidence (GRADE)	Plain language summary
	Risk with intravenous fluid resuscitation	Risk with oral/enteral fluid resuscitation				
**Mortality** [in-hospital]	143 per 1,000	**181 per 1,000** (52 to 472)	**OR 1.33** (0.33 to 5.36)	70 (2 RCTs)	⊕◯◯◯ Very low Due to serious risk of bias^1^, indirectness^2^ and imprecision^3^	Oral/enteral fluid resuscitation may increase in-hospital mortality compared to IV fluid resuscitation, but the available evidence is very uncertain.
**Acute kidney injury** [within 72 h post-burn injury]	0 per 1,000	**0 per 1,000** (0 to 0)	not estimable	24 (1 RCT)	⊕◯◯◯ Very low Due to serious risk of bias^1^, indirectness^2^ and imprecision^3^	The effect of oral/enteral fluid resuscitation on the occurrence of acute kidney injury within 72 h post-major burn injury is not estimable from available evidence.
**Urine output** [during first 24 h post-burn injury]	−	SMD **0.17 SD lower** (0.65 lower to 0.31 higher)	−	70 (2 RCTs)	⊕⊕⊕◯ Moderate Due to indirectness^2^	Oral/enteral fluid resuscitation likely results in little to no difference in urine output during the first 24 h post-major burn injury.
**Fluid over-resuscitation** − not reported	−	−	−	−	−	
**Aspiration events** − not reported	−	−	−	−	−	
*The risk in the intervention group (and its 95% confidence interval) is based on the assumed risk in the comparison group and the **relative effect** of the intervention (and its 95% CI). **CI:** confidence interval; **OR:** odds ratio; **SMD:** standardised mean difference						

***Explanations***

1. Risk of bias: serious. Overall high risk due to high risk of bias in predominant study, which had significant deviations from intended intervention with 9/15 (60%) of oral fluid (intervention) group crossing to IV fluids due to gastrointestinal intolerance such as nausea or vomiting.

2. Indirectness: serious. Findings from clinical trial setting of individual burn care may not be directly applicable to setting of mass casualty incidents, which likely have (1) less clinical attention per patient, (2) greater proportion of delayed presentations or interventions, and (3) include patients with multiple injuries, inhalational injuries, and comorbidities, who were excluded for some of these trials.

3. Imprecision: serious. Few total events. Result with wide confidence interval which includes both no effect and large effect.

## 5 Results from animal studies

### 5.1 Study characteristics

All included animal studies were from the last 25 years, and were conducted in either the United States (n=4) or China (n=4). Four studies were conducted with pigs [10], [11], [28], [30], three with dogs [31], [32], [33] and one with rats [29]. Thermal burn severity in these experiments ranged from 35 to 50% TBSA. All studies were controlled experiments with random division of groups, and all contained multiple intervention and/or comparison groups. Three studies [11], [31], [32] included comparison of enteral fluid resuscitation (with ORS or hypertonic saline delivered via gastric or duodenal infusion or manual gavage) versus IV (Ringer’s lactate or 0.9% NaCl + 5% glucose solution given according to Parkland formula). Five studies [10], [29], [30], [31], [33] included comparison of enteral versus no fluid resuscitation. Some studies had additional study groups, such as experimental additives/adjuncts to ORS, low volume ORS and/or ad libitum water access; these additional study groups were not included in meta-analyses to avoid clinical heterogeneity.

### 5.2 Risk of bias within studies

**Fig. 4:**
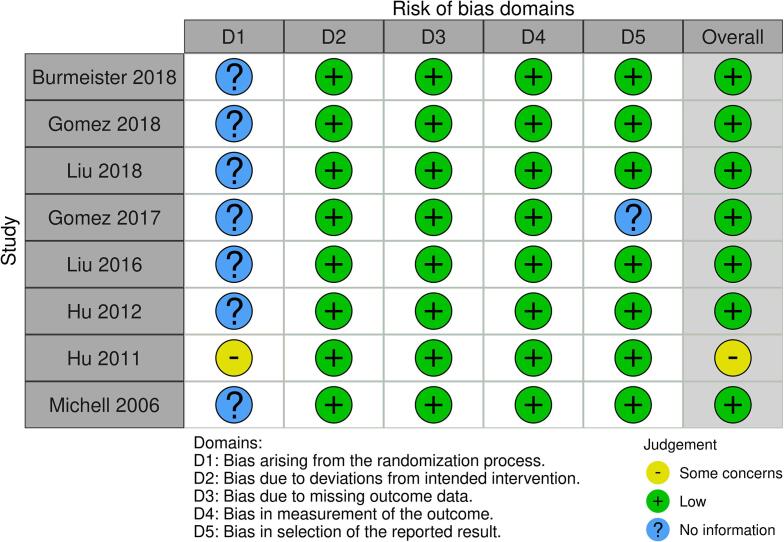
Assessed risk of bias within controlled studies (animal studies) using RoB 2 tool.

The assessed risk of bias within the animal studies is presented in Fig. 4.

The overall risk of bias was low across the animal studies with the limitation that studies only stated randomization or random division into groups with no further information on the process to allow assessment of this domain.

### 5.3 Effects of enteral versus IV fluid resuscitation

**Fig. 5:**
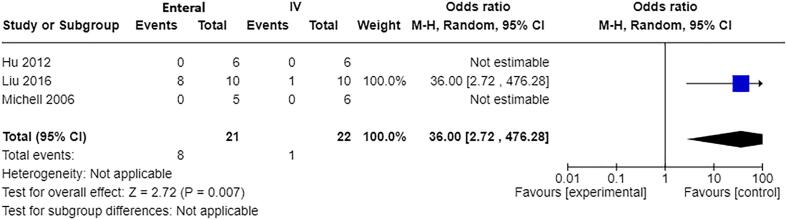
Forest plot of enteral versus IV fluids (animal studies) for outcome: Mortality (within study time frame).

Mortality: Three studies [11], [31], [32] with a total of 43 participants contributed to the meta-analysis of effect on mortality (Fig. 5). However, two of the studies had zero events in both intervention and comparison groups, which left one study with estimable OR. This showed increased mortality at 48 h with enteral fluid resuscitation compared to IV (OR 36.00, 95% CI 2.72–476.28, p=0.007).

AKI: Only one study [31] with 14 participants contained data pertaining to AKI. Renal function, as indicated by mean serum creatine level, was worse with enteral fluid resuscitation (Cr 83 +/- 8 mmol/L) compared to IV fluid resuscitation (Cr 61 +/- 6 mmol/L) at 24 h post-burn injury with a mean difference of 22 mmol/L (95% CI 15.8–28.2, p<0.01).

Urine output: Only one study [11] with 11 participants contained comparative data on urine output. At 4.5 h post-burn injury (duration of study), mean urine output was higher with enteral fluid resuscitation (1.3 +/- 0.5 ml/kg/hr) compared to IV (0.3 +/- 0.1 ml/kg/hr) (mean difference 1.0 ml/kg/hr, 95% CI 0.55–1.45 ml/kg/hr, p<0.01).

Complications of fluid over-resuscitation: No data to estimate effect.

Aspiration events: No data to estimate effect.

### 5.4 Effects of enteral versus no fluid resuscitation

Mortality: Four studies [10], [29], [31], [33] with a total of 90 participants contributed to the meta-analysis on mortality (Fig. 6A). Mortality at 24 h post-burn injury was reduced with enteral fluid resuscitation compared to no fluid resuscitation (OR 0.29, 95% CI 0.08–1.09, p=0.07). No substantial heterogeneity was indicated (I^2^=0%), although the studies involved difference species.

**Fig. 6A:**
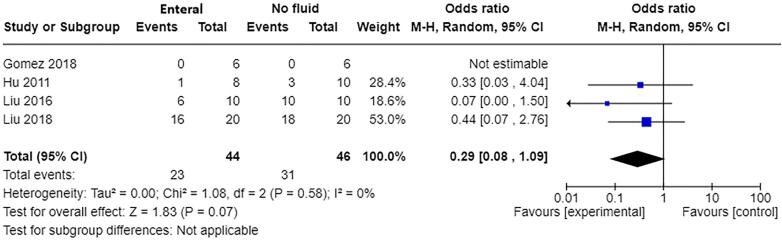
Forest plot of enteral versus no fluids (animal studies) for outcome: Mortality (at 24 h post-burn injury).

AKI: Two studies [10], [31] with a total of 32 participants contributed to the meta-analysis on AKI (Fig. 6B). Mean serum creatinine levels at 48 h post-burn injury were better with enteral fluid resuscitation compared to no fluid resuscitation (standard mean difference −3.48, 95% CI −4.69 to −2.28, p<0.01).

**Fig. 6B:**
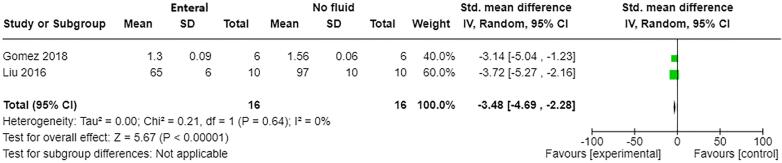
Forest plot of enteral versus no fluids (animal studies) for outcome: AKI (mean serum creatinine at 48 h post-burn injury).

Urine output: One study [10] with 12 participants provided comparative data on urine output. Mean urine output over the first 48 h post-burn injury was 0.55 ml/kg/hr higher (95% CI 0.38–0.72 ml/kg/hr, p<0.01) with enteral fluid resuscitation (1.07 +/- 0.21 ml/kg/hr) compared to no fluid resuscitation (0.52 +/- 0.03 ml/kg/hr).

Complications of fluid over-resuscitation: No data to estimate effect.

Aspiration events: No data to estimate effect.

### 5.5 Certainty of evidence

Using the GRADE approach, the certainty of evidence for the effect estimates derived from animal studies was rated as low to very low across comparisons and outcomes. Downgrading due to very serious indirectness was applicable across the board owing to the limitations of findings from laboratory-based, animal studies for human patients in real-world clinical settings, not least in contexts of mass casualties. Further downgrading of certainty for some effect estimates occurred because of serious imprecision from few events and/or small sample size. Summaries of findings from animal studies on enteral versus IV fluids and on enteral versus no fluids are presented in Table 4 and Table 5, respectively.

**Table 4:** Summary of Findings table for enteral compared to IV fluid resuscitation for initial management of major burns (animal studies).

Outcomes	Anticipated absolute effects* (95% CI)		Relative effect (95% CI)	№ of participants (studies)	Certainty of the evidence (GRADE)	Plain language summary
	Risk with intravenous fluid resuscitation	Risk with enteral fluid resuscitation				
**Mortality** [within study timeframe]	45 per 1,000	**632 per 1,000** (115 to 958)	**OR 36.00** (2.72 to 476.28)	43 (3 RCTs)	⊕◯◯◯ Very low Due to very serious indirectness^1^ and serious imprecision^2^	Based on animal studies, enteral fluid resuscitation compared to intravenous fluid resuscitation may increase mortality, but the available evidence is very uncertain.
**Acute kidney injury** [serum creatinine level at 24 h post-burn injury]	Mean serum creatinine level at 24 h post-burn injury was **61** mmol/L	MD **22 mmol/L higher** (15.8 higher to 28.2 higher)	−	20 (1 RCTs)	⊕◯◯◯ Very low Due to very serious indirectness^1^ and serious imprecision^3^	Based on animal studies, enteral fluid resuscitation compared to intravenous fluid resuscitation may result in worse serum creatinine levels at 24 h post-major burn injury, but the available evidence is very uncertain
**Urine output at end of study** [4.5 h post-burn injury]	Mean urine output at end of study [4.5 h post-burn injury] was **0.3** ml/kg/h	MD **1 ml/kg/h higher** (0.55 higher to 1.45 higher)	−	11 (1 RCT)	⊕◯◯◯ Very low Due to very serious indirectness^1^ and serious imprecision^3^	Based on animal studies, enteral fluid resuscitation compared to intravenous fluid resuscitation may increase urine output during the first 4.5 h post-major burn injury, but the available evidence is very uncertain.
Fluid over-resuscitation − not reported	−	−	−	−	−	
Aspiration events − not reported	−	−	−	−	−	
*The risk in the intervention group (and its 95% confidence interval) is based on the assumed risk in the comparison group and the **relative effect** of the intervention (and its 95% CI). **CI:** confidence interval; **MD:** mean difference; **OR:** odds ratio; **SMD:** standardised mean difference						

***Explanations***

1. Indirectness: very serious. Laboratory-based study using animal models therefore significant indirectness in applicability to (1) human patients in (2) real-world clinical setting with (3) mass casualties.

2. Imprecision: serious. Few events. Result with wide confidence interval.

3. Imprecision: serious. Single study with small sample size. Optimal information size not met.

**Table 5:** Summary of Findings table for enteral compared to no fluid resuscitation for initial management of major burns (animal studies).

Outcomes	Anticipated absolute effects* (95% CI)		Relative effect (95% CI)	№ of participants (studies)	Certainty of the evidence (GRADE)	Plain language summary
	Risk with no fluids	Risk with enteral fluid resuscitation				
**Mortality** [at 24 h post-burn injury]	674 per 1,000	**375 per 1,000** (142 to 693)	**OR 0.29** (0.08 to 1.09)	90 (4 RCTs)	⊕◯◯◯ Very low Due to very serious indirectness^1^ and serious imprecision^2^	Based on animal studies, enteral fluid resuscitation compared to no fluid resuscitation may reduce mortality at 24 h post-major burn injury, but the available evidence is very uncertain.
**Acute kidney injury** [serum creatinine level at 24 h post-burn injury]	−	SMD **3.48 SD lower** (4.69 lower to 2.28 lower)	−	32 (2 RCTs)	⊕⊕◯◯ Low Due to very serious indirectness^1^	Based on animal studies, enteral fluid resuscitation compared to no fluid resuscitation may result in better serum creatinine levels at 24 h post-major burn injury.
**Urine output** [during first 48 h post-burn injury]	Mean urine output [first 48 h post-burn injury] was **0.52** ml/kg/h	MD **0.55 ml/kg/h higher** (0.38 higher to 0.72 higher)	−	12 (1 RCT)	⊕◯◯◯ Very low Due to very serious indirectness^1^ and serious imprecision^3^	Based on animal studies, enteral fluid resuscitation compared to no fluid resuscitation may increase urine output during the first 48 h post-major burn injury, but the available evidence is very uncertain.
Fluid over-resuscitation − not reported	−	−	−	−	−	
Aspiration events − not reported	−	−	−	−	−	
*The risk in the intervention group (and its 95% confidence interval) is based on the assumed risk in the comparison group and the **relative effect** of the intervention (and its 95% CI). **CI:** confidence interval; **MD:** mean difference; **OR:** odds ratio; **SMD:** standardised mean difference						

***Explanations***

1. Indirectness: very serious. Laboratory-based study using animal models therefore significant indirectness in applicability to (1) human patients in (2) real-world clinical setting with (3) mass casualties.

2. Imprecision: serious. Wide confidence interval that includes no effect

3. Imprecision: serious. Single study with small sample size. Optimal information size not met.

## 6 Discussion

The purpose of this review was to synthesize the available evidence on oral/enteral fluid resuscitation as compared to IV and to no fluid resuscitation for patients with major burns, and to assess the strength of this evidence to help inform updated WHO recommendations on burns care in MCIs. The review found a paucity of studies and data comparing the effect of oral/enteral fluid resuscitation to IV or no fluid resuscitation in major burns for the key clinical outcomes of interest. The evidence that is available is very uncertain, but suggests that oral/enteral fluid resuscitation may be associated with increased mortality and reduced renal function compared to IV resuscitation. More certain is the effect on urine output, for which there is probably little to no difference with oral/enteral fluid resuscitation compared to IV resuscitation. Conversely, when compared to no fluid resuscitation, enteral fluids significantly improve mortality, renal function, and urine output. However, this is based solely on animal studies so the certainty of evidence remains very low. Lastly, the review found no data to determine effect on aspiration events, a potential harm introducible by oral/enteral resuscitation, or on fluid over-resuscitation, for which oral/enteral resuscitation has been proposed as a safety advantage, especially in MCIs where safe monitoring and titration of IV fluids may be difficult [3], [9].

In short, the relative safety and efficacy of oral/enteral fluid resuscitation compared to standard IV resuscitative care remains undetermined, but oral/enteral fluids may be better than no fluids when IV fluid resuscitation is unavailable or significantly delayed, such as possible in MCIs. The way in which this may be applied in practice will depend on additional contextual factors and considerations. For example, MCIs vary in number of casualties, types and severity of injuries, time and distance to medical assistance, degree to which staff, supplies and spaces are overwhelmed, and capacity of local surge systems to mitigate. These contextual factors will also influence risk–benefit for oral/enteral fluid resuscitation. Additionally, unlike the binary nature of this review question, consideration may also be given to instituting oral/enteral fluid resuscitation in conjunction with IV fluids. This approach was applied and showed feasibility in three studies [24], [25], [26] that were identified by the review but excluded in main analysis as their results did not distinctly compare oral/enteral to IV fluid resuscitation. In MCIs, combined oral/enteral and IV fluid resuscitation may be used to reduce individual IV fluid requirements so that a greater number of people may be treated and/or to conserve IV fluid supplies and expertise for those not amenable to oral fluids or those with greatest need. The small study by *Monafo* found that oral fluids could replace up to 77.5% of IV fluid requirements for major burns [24]. There is also potential for synergistic connection between oral/enteral fluid resuscitation and early enteral nutrition and feeding, which has been recommended as important for improving gut blood flow and function, and for reducing negative pathophysiologic changes, such as bacterial translocation, following major burn injury [7], [34], [35].

The question of amenability to oral/enteral fluids requires further consideration. Sub-group analyses were ultimately not feasible in this review. However, as indicated by the WHO GDG, there may be clinically important sub-groups for which risk–benefit of oral/enteral fluid resuscitation differs. Clinical common sense would suggest that patients with oral/facial burns, concomitant severe trauma, altered consciousness or overt shock would not be amendable to oral fluid resuscitation. Some of these caveats are already seen in various recommendations that mention oral/enteral fluid resuscitation [1], [2], [3], [6]. Children, particularly infants, may also be less able to tolerate oral/enteral fluids when acutely unwell. Additionally, efficacy limits of oral/enteral fluid resuscitation in terms of burn severity remains uncertain. Animal studies have indicated that intestinal absorption capacity is sufficient to resuscitate burns up to 40% TBSA [11]. However, this may become insufficient with more severe burns where there are increasing fluid requirements while gastrointestinal function simultaneously becomes more impaired [10], [33]. Many guidelines limit their recommendations for oral/enteral fluid resuscitation to burns ≤40% TBSA [3], [6], [8], [9]. Notably in this review, all animal and human studies that contributed to the effect estimates had study populations with burns ≤55% TBSA.

Finally, while there are clear logistical and resource advantages of oral/enteral fluid resuscitation over IV in MCIs, such as lower volume and weight of ORS sachets versus IV fluid bags, less equipment and less expertise for administration [9], [36], its implementation is not without resource requirements: Patients need to have access to clean potable water and ORS sachets (or equivalent salt-containing solutions); those requiring assistance for oral fluid administration, such as the very young, the elderly and those with disabilities and/or injuries, need to be identified and supported; and monitoring and evaluation of clinical response and tolerance are still required.

Further studies are certainly needed to better determine the effect of oral/enteral versus IV fluid resuscitation for major burns, and to strengthen certainty of evidence. There are at least two RCTs [37], [38] on this question currently underway, the results of which may contribute in this regard. In addition to establishing overall safety and efficacy, research is also required to delineate efficacy limits, understand any differing effects for clinically important sub-groups, and identify optimal formulation(s) and regimens for oral/enteral fluid resuscitation [1], [2], [13].

### 6.1 Limitations

Despite a comprehensive search strategy, there may have been relevant studies that were missed in this review. Within this, the key limitation was exclusion of studies that were not written in English. A post-hoc assessment was conducted to understand the potential types of studies and data missed due to this exclusion by language. Of the 10 such excluded studies, one was a knowledge review article (wrong article type), eight were animal studies, and one was a human clinical study from 1989 for which the full text could not be retrieved. However, the search strategy did identify studies from non-indexed publications as well as two in-progress RCTs, of which the pilot results of one [23] was included in the analyses. An updated review incorporating complete results would be warranted once these studies have concluded.

In addition to limitations from the small number studies and participants that contributed to effect estimates, the applicability of these estimates is also constrained by limitations in design of included studies. In particular, the human study populations that contributed to effect estimates were limited to those with less than 55% TBSA burns, and largely excluded participants with comorbidities, associated injuries, inhalational burns and pregnancy, among other conditions, which may not reflect the likely patient population and case-mix expected during an MCI.

Finally, as already alluded, the structure of this review question presents oral/enteral versus IV (or no fluid resuscitation) as binary choices. However, in reality, combination approaches, such as supplementing IV fluids with oral fluids, could also be reasonably considered and feasibly utilized.

## 7 Conclusions

Current available evidence on oral/enteral fluid resuscitation for major burns is limited and of low certainty. IV fluid resuscitation remains standard of care and is the preferred route whenever possible. However, in situations where IV fluid resuscitation is not achievable or likely to be significantly delayed, such as during an overwhelming MCI, oral/enteral fluid resuscitation could be considered.

## 8 Disclaimer

The authors alone are responsible for the views expressed in this article and they do not necessarily represent the views, decisions or policies of the institutions with which they are affiliated.

## 9 Funding sources

This research did not receive any specific grant from funding agencies in the public, commercial, or not-for-profit sectors.

## Declaration of competing interest

The authors declare that they have no known competing financial interests or personal relationships that could have appeared to influence the work reported in this paper.

## Figures and Tables

**Table:** 

**#**	**Searches**	**Results**
1	“Burns”[Mesh] OR “burn units”[mesh] OR burn[ti] OR burns[ti] OR postburn*[ti] OR thermally-injured[ti] OR thermal-injur*[ti]	68,921
2	“Rehydration Solutions”[mh] OR “Fluid Therapy”[mh] OR “Plasma Volume”[mh] OR “Resuscitation”[Mesh] OR fluid*[tiab] OR hydration[tiab] OR rehydration[tiab] OR resuscitat*[tiab]	763,444
3	oral[tiab] OR ORS[tiab] OR enteral[tiab]	4,496
4	#1 AND #2 AND #3	199
5	enteral-nutrition*[ti] OR feeding[ti]	59,480
6	#4 NOT #5	180

## Appendix . Example search strategy.

Database: Pubmed

URL: https://www.pubmed.gov

Date Searched: 08.09.2023
